# Supplementary material for: Foundation neural-networks quantum states as a unified Ansatz for multiple hamiltonians
Source: Nat Commun. 2025 Aug 5;16:7213. doi: 10.1038/s41467-025-62098-x (PMC12325958; doi:10.1038/s41467-025-62098-x)
Supplement: Supplementary file 1 — Supplementary Information pdf [file 41467_2025_62098_MOESM1_ESM.pdf]

Supplementary Information: Foundation Neural-Networks Quantum States as a Unified Ansatz for Multiple Hamiltonians

Hyperparameters

In Supplementary Table 1 we provide the hyperparameters of the FNQS architecture and the optimization protocol used to study the various systems. See Refs. [1, 2, 3] for more details about the role of the different hyperparameters.

Systematic energy improvement

In this section, we systematically increase the expressivity of the FNQS by varying the number of parameters and assess the resulting performance using the V-score [4], a metric known to correlate with the accuracy of the wave function. The V-score for the system $\hat{H}_{\gamma}$ is defined as

$$\text{V-score}(\boldsymbol{\gamma}) = N \frac{\langle \hat{H}_{\gamma}^2 \rangle_{\gamma} - \langle \hat{H}_{\gamma} \rangle_{\gamma}^2}{\langle \hat{H}_{\gamma} \rangle_{\gamma}^2} \ . \tag{1}$$

A key advantage of the V-score is that it can be computed without requiring access to exact reference energies, making it particularly suitable for benchmarking variational methods on large systems [4].

The goal of this analysis is to demonstrate that the accuracy of the FNQS can be systematically improved by increasing the size of the neural network, which we control by adjusting the number of layers. We carry out this study on the J_1–J_2 Heisenberg model on a $L \times L$ square lattice, described by the Hamiltonian (with periodic boundary conditions):

$$\hat{H} = J_1 \sum_{\langle \boldsymbol{r}, \boldsymbol{r}' \rangle} \hat{\boldsymbol{S}}_{\boldsymbol{r}} \cdot \hat{\boldsymbol{S}}_{\boldsymbol{r}'} + J_2 \sum_{\langle\langle \boldsymbol{r}, \boldsymbol{r}' \rangle\rangle} \hat{\boldsymbol{S}}_{\boldsymbol{r}} \cdot \hat{\boldsymbol{S}}_{\boldsymbol{r}'} \ . \tag{2}$$

Specifically, we consider system sizes ranging from $L = 6$ to $L = 12$, and vary the number of layers of the neural network architecture from $n_l = 2$ to $n_l = 8$. The FNQS is optimized

	Architecture				Optimization			
	n_l	n_h	d	b	M	N_{opt}	η	λ
Ising trasverse field	6	12	72	4	10000	2000	0.03	10^{-4}
J_1-J_2-J_3 Heisenberg	8	12	72	2×2	16000	3500	0.03	5×10^{-4}
Random transverse field Ising	6	12	72	4	10000	4000	0.03	10^{-4}

Supplementary Table 1: This table presents the hyperparameters of the FNQS wave function used to simulate different systems. The *Architecture* columns specify the number of layers n_l, number of heads n_h, embedding dimension d, and patch size b. The *Optimization* columns list the hyperparameters for the Stochastic Reconfiguration method, including the total batch size M, the number of optimization steps N_{opt}, the learning rate η, and the diagonal shift regularization λ.

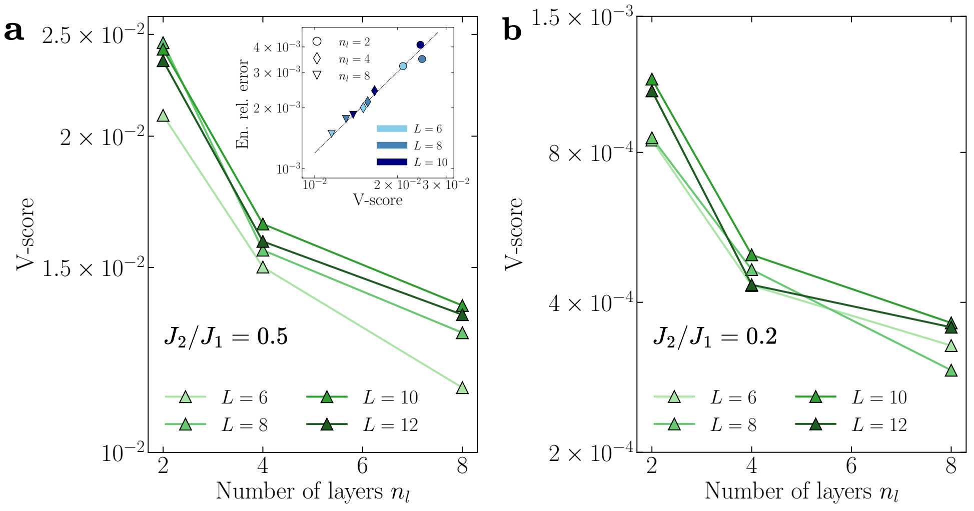

Supplementary Figure 1: V-score for the J_1–J_2 Heisenberg model on the square lattice as a function of the number of layers n_l of the FNQS, for increasing the system size from $L = 6$ to $L = 12$. Panel (a) shows results for the highly frustrated point $J_2/J_1 = 0.5$, while panel (b) corresponds to the less frustrated point $J_2/J_1 = 0.2$. The inset in panel (a) displays the correlation between the V-score and the relative energy error at $J_2/J_1 = 0.5$ for system sizes $L = 6$ to $L = 10$. Reference energies for $L = 8$ and $L = 10$ are obtained via zero-variance extrapolation from Refs. [5, 6].

across $\mathcal{R} = 1000$ coupling values, uniformly sampled in the interval $J_2/J_1 \in [0, 1]$. Importantly, for a fixed network architecture and total number of Monte Carlo samples M, the computational cost of each simulation remains independent of $\mathcal{R}$. Additionally, while the computational complexity of the ViT scales quadratically with the input sequence length, this cost can be mitigated through various strategies [2].

After optimizing the FNQS, we focus on two representative values of the frustration ratio: $J_2/J_1 = 0.5$ and $J_2/J_1 = 0.2$. In panel (a) of Supplementary Figure 1, we present results for $J_2/J_1 = 0.5$, a highly frustrated point in the phase diagram [7]. At this value, high-precision ground-state energy estimates obtained via zero-variance extrapolation are available for system sizes beyond $L = 6$ [5, 6], enabling us to validate the correlation between the relative energy error and the V-score, as shown in the inset of panel (a) of Supplementary Figure 1. In panel (b) of Supplementary Figure 1, we report the V-score for $J_2/J_1 = 0.2$, a less frustrated point closer to the unfrustrated Heisenberg model. Due to the reduced complexity of the ground state in this region, the V-scores are systematically lower compared to the highly frustrated case at $J_2/J_1 = 0.5$. By analyzing system sizes up to $N = 144$ spins for both frustration ratios, we find that the V-score, and thus the accuracy, systematically improves when increasing the number of parameters in the network, while marginally deteriorating with the system size for a fixed architecture. These results highlight the size-consistency of the ViT parametrization of the FNQS, even when the model is simultaneously optimized across multiple systems. We remark that further improvements in accuracy are achievable by explicitly incorporating the symmetries of the underlying Hamiltonian, although this lies beyond the scope of the present analysis.

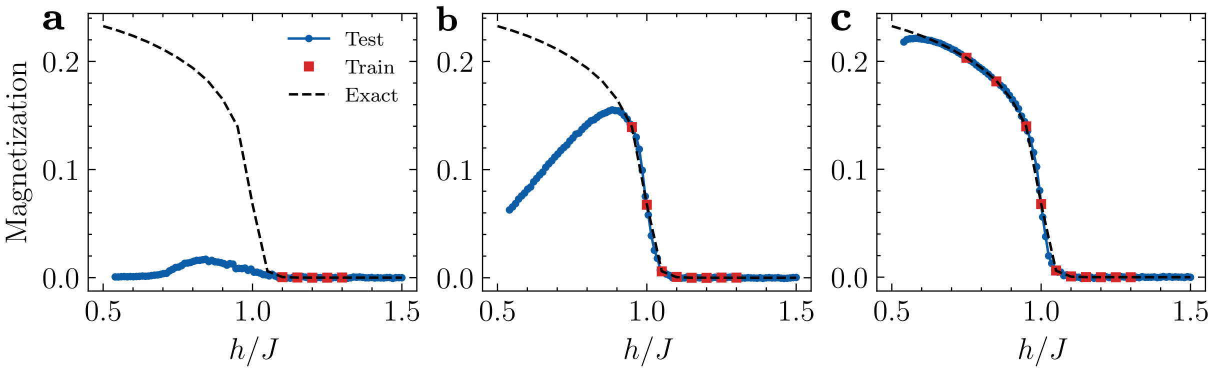

Supplementary Figure 2: Generalization of FNQS across a quantum phase transition in the transverse field Ising chain. **Panel a:** The FNQS is trained exclusively in the disordered phase ($h > 1.2$) and evaluated across a range of transverse field strengths. **Panel b** and **Panel c**: Systematic improvement in generalization is observed as instances from the ordered phase are gradually introduced into the training set.

Generalization across phase boundaries

In this section, we investigate the generalization properties across phase boundaries of FNQS using the transverse field Ising chain (see the "Results" section for the model definition). We train the model exclusively in the disordered phase, for transverse field strengths $h/J > 1.2$, and evaluate its performance across the phase transition, with particular focus on the ordered regime at $h/J < 1.0$. As shown in panel (a) of Supplementary Figure 2, a model trained solely in the disordered phase fails to accurately capture the ground-state properties in the ordered phase. However, its performance improves systematically when instances from the ordered phase are incorporated into the training set [refer to panels (b) and (c)].

This simple example shows that, in general, FNQS are not expected to extrapolate across phase boundaries, as the physical properties change significantly across different phases. This limitation is not unique to FNQS, but rather a fundamental constraint of machine learning approaches in general. As with language models, where a model trained on a set of languages cannot be expected to understand an additional one, FNQS cannot generalize to physical regimes absent from the training distribution. For successful generalization, the training set must adequately represent the diversity of the target distribution (see the section "Out-of-distribution generalization" in the main text).

References

[1] Riccardo Rende, Luciano Loris Viteritti, Lorenzo Bardone, Federico Becca, and Sebastian Goldt. A simple linear algebra identity to optimize large-scale neural network quantum states. *Communications Physics*, 7(1), August 2024.

[2] Luciano Loris Viteritti, Riccardo Rende, Alberto Parola, Sebastian Goldt, and Federico Becca. Transformer wave function for two dimensional frustrated magnets: Emer-

gence of a spin-liquid phase in the shastry-sutherland model. *Phys. Rev. B*, 111:134411, Apr 2025.

[3] F. Becca and S. Sorella. *Quantum Monte Carlo Approaches for Correlated Systems.* Cambridge University Press, 2017.

[4] Dian Wu, Riccardo Rossi, Filippo Vicentini, Nikita Astrakhantsev, Federico Becca, Xiaodong Cao, Juan Carrasquilla, Francesco Ferrari, Antoine Georges, Mohamed Hibat-Allah, Masatoshi Imada, Andreas M. Läuchli, Guglielmo Mazzola, Antonio Mezzacapo, Andrew Millis, Javier Robledo Moreno, Titus Neupert, Yusuke Nomura, Jannes Nys, Olivier Parcollet, Rico Pohle, Imelda Romero, Michael Schmid, J. Maxwell Silvester, Sandro Sorella, Luca F. Tocchio, Lei Wang, Steven R. White, Alexander Wietek, Qi Yang, Yiqi Yang, Shiwei Zhang, and Giuseppe Carleo. Variational benchmarks for quantum many-body problems. *Science*, 386(6719):296–301, 2024.

[5] Wen-Jun Hu, Federico Becca, Alberto Parola, and Sandro Sorella. Direct evidence for a gapless Z_2 spin liquid by frustrating néel antiferromagnetism. *Phys. Rev. B*, 88:060402, Aug 2013.

[6] Ao Chen and Markus Heyl. Empowering deep neural quantum states through efficient optimization. *Nature Physics*, 20(9):1476–1481, 2024.

[7] Y. Nomura and M. Imada. Dirac-type nodal spin liquid revealed by refined quantum many-body solver using neural-network wave function, correlation ratio, and level spectroscopy. *Phys. Rev. X*, 11:031034, Aug 2021.
